# Sex-dependent role of Neuropeptide-S on anxiety, fear conditioning, and alcohol seeking in alcohol preferring rats

**DOI:** 10.1016/j.neuropharm.2025.110598

**Published:** 2025-07-23

**Authors:** Min Li, Sara De Carlo, Laura Soverchia, Scott P. Runyon, Stewart Clark, Marisa Roberto, Carolina L. Haass-Koffler, Roberto Ciccocioppo, Douglas J. Sheffler, Nazzareno Cannella

**Affiliations:** a School of Pharmacy, Center for Neuroscience, Pharmacology Unit, University of Camerino, Italy; b Research Triangle Institute, Center for Drug Discovery, Research Triangle Park, NC, 27709, USA; c Department of Pharmacology and Toxicology, State University of New York at Buffalo, Buffalo, NY, USA; d Department of Translational Medicine, The Scripps Research Institute, La Jolla, CA, USA; e Center for Alcohol and Addiction Studies, Department of Psychiatry and Human Behavior, Department of Behavioral and Social Sciences, Carney Institute for Brain Sciences, Brown University, Providence, RI, USA; f Center for Therapeutics Discovery, Sanford Burnham Prebys Medical Discovery Institute, La Jolla, CA, USA

**Keywords:** Alcoholism, Anxiety, Stress, Post-traumatic stress disorder, Sex-differences, Genetic predisposition to AUD, Neuropeptide S

## Abstract

Alcohol Use Disorder (AUD) is a global health concern, with stress playing a crucial role in its development and persistence. Currently, no pharmacotherapies specifically targeting stress are approved for AUD treatment. Neuropeptide S (NPS) plays a dual role in stress regulation, exhibiting both anxiolytic and stress-enhancing effects. While NPS reduces alcohol self-administration (ASA) in alcohol preferring rats, its role in AUD-related stress and anxiety remains unclear.

This study investigated the behavioral effects of NPS in male and female Marchigian Sardinian alcohol-preferring (msP) rats. To assess its impact on locomotion, anxiety, and fear memory, we conducted an open-field, an elevated plus maze (EPM), and a fear conditioning (FC) paradigm following intracerebroventricular administration of NPS. Furthermore, we examined the effects of NPS on ASA and yohimbine-induced reinstatement of alcohol-seeking in msP rats.

Our results indicate that NPS administration increased locomotor activity in both sexes and selectively alleviated generalized anxiety levels in male rats in the EPM test. In the FC task, administration of NPS immediately after FC test facilitated the extinction of fear memories in females but not in males. Notably, NPS reduced ASA in both female and male rats but did not alter yohimbine-induced reinstatement of alcohol-seeking. In conclusion, NPS modulates anxiety in a sex-dependent manner. Since both alcohol and NPS alleviate anxiety and fear conditioning in msP rats, NPS may reduce alcohol intake by replacing the anxiolytic properties of alcohol. These effects appear to be sex-dependent, with NPS primarily alleviating generalized anxiety in males and facilitating fear extinction in females.

## 1. Introduction

Alcohol use disorder (AUD) is a global crisis, contributing to nearly three million deaths annually, accounting for 5 % of all deaths worldwide. A substantial proportion of fatalities from cardiovascular diseases, malignant neoplasms, digestive diseases, and both unintentional and intentional injuries are linked to alcohol consumption (WHO, 2024).

Stress is a major player in the trajectory to AUD. Stress-related psychiatric conditions, such as anxiety (Anker et al., 2019) and post-traumatic stress disorder (PTSD) (Sonne et al., 2003) are often co-morbid with AUD. While both men and women consume alcohol to cope with affective negative states, stress seems to be a stronger driver of alcohol drinking in women than men (Peltier et al., 2019). For example, women are more likely than men to be diagnosed with co-morbid AUD-PTSD (Sonne et al., 2003), women with AUD exhibit higher levels of anxiety (King et al., 2003), and they report a higher frequency of heavy alcohol drinking to cope with negative emotional states (Abulseoud et al., 2013). Despite the pivotal role of stress in AUD, no pharmacotherapies specifically targeting the stress system has been approved to date (Cannella et al., 2019; Schank et al., 2012). Although the extensive translational efforts (Haass-Koffler and Sheffler, 2023), unfortunately, stress-targeting drugs that reached clinical trials failed to show efficacy in humans (Kwako et al., 2015; Schwandt et al., 2016). Therefore, it is paramount to validate new pharmacological targets that interact with the role of the stress system in these psychiatric conditions.

The Neuropeptide S (NPS) is a brainstem neuromodulator whose receptor (NPSR) is expressed throughout the brain (Xu et al., 2007). NPS is the only known endogenous compound that promotes arousal and activates the stress system, and yet it is anxiolytic at the same doses (Cannella et al., 2013; Smith et al., 2006; Xu et al., 2004). For instance, NPS has been shown to activate the hypothalamic-pituitary-adrenal axis (Smith et al., 2006), reduce anxiety (Rizzi et al., 2008; Wegener et al., 2012) and facilitate fear extinction in a fear conditioning test (Jungling et al., 2008, 2015), a model of PTSD.

NPS also plays a dual role in alcohol-seeking behavior. Exogenous administration of NPS promoted relapse-like behavior in non-preferring Wistar rats (Cannella et al., 2009; Ubaldi et al., 2016), while it reduced alcohol self-administration in both Marchigian Sardinian alcohol preferring (msP) male (Cannella et al., 2016) and Indiana female (Badia-Elder et al., 2008) rats. The msP rat line is characterized by innate negative affective states. The increased alcohol preference is considered a coping strategy in which alcohol is consumed to relieve these negative affective states (Borruto et al., 2021; Ciccocioppo et al., 1999, 2006; Hansson et al., 2006, 2007; Natividad et al., 2017; Vozella et al., 2021, 2023).

In this study, we aimed to examine the stimulatory, anxiolytic, and stress-related role of NPS in male and female msP rats and to evaluate possible links with their innate high alcohol drinking. First, we tested whether NPS retains its stimulatory effect in an open-field test, followed by assessment of its effects on generalized anxiety and consolidation of fear memories in an elevated plus maze (EPM) and fear conditioning (FC) test, respectively. Since anxiety and PTSD are often co-morbid with AUD, we then confirmed the ability of NPS to reduce alcohol self-administration in male msP rats and investigated whether this neuromodulator retains the same effect in female msP rats as well. Finally, in previous studies, we demonstrated that NPS can both reinstate alcohol seeking on its own and exacerbate cued reinstatement in Wistar rats by activating stress-related circuitries; however, these relapse-promoting effects were not observed in msP rats (Cannella et al., 2009, 2016; Ubaldi et al., 2016). Given that stress is also a trigger of relapse, we sought to expand on our previous findings by testing the hypothesis that NPS does not exacerbate yohimbine-induced reinstatement of alcohol seeking behavior (Curley et al., 2022).

## 2. Materials and methods

### 2.1. Animals

Male and female msP rats bred at the University of Camerino (96th generation) were aged 9 weeks at the beginning of experimental procedures. Rats were housed in group cages in a room with reversed 12 h/12 h light/dark cycle (lights off at 8:30 a.m.), constant temperature (20–22 °C), and humidity (45–55 %). Males and females were housed in separate rooms. Food (4RF18, Mucedola, Italy) and tap water were available *ad libitum*. All procedures were conducted during the dark phase of the light/dark cycle and were in adherence with *Animals (Scientific Procedures) Act 1986 and associated guidelines*, the *European Council Directive EU Directive 2010/63 for the Care and Use of Laboratory Animals* and the *National Institutes of Health Guide for the Care and Use of Laboratory Animals*. All animal experiments complied with ARRIVE (Animal Research: Reporting of In Vivo Experiments) guidelines.

### 2.2. Surgeries

Rats were surgically implanted with an intracerebroventricular (ICV) cannula under isoflurane anaesthesia (1.5–2.5 %). Guide cannulae were stereotaxically implanted and cemented to the skull. Coordinates referred to bregma were as follows: antero-posterior: −1.0 mm; medio-lateral: −1.8 mm; dorso-ventral: −2.0 mm. To prevent postoperative pain, rats were treated subcutaneously with 0.1 ml of meloxicam (5 mg/ml). Surgeries were followed by a one-week recovery period, during which rats were left undisturbed in their home cages. The antibiotic Enrofloxacin (Baytril ^®^) was diluted in the drinking water (50 mg/100 ml) for five days following surgery.

Cannula placement was verified before experiments by ICV injection of 100 ng of angiotensin II; only animals showing a clear dipsogenic response (consumption of at least 5 ml of water within 5 min) were used for further experimentation.

### 2.3. Drug injection

NPS (Sigma-Aldrich) was dissolved in sterile isotonic saline and administered ICV in a volume of 1 μl/rat using a stainless-steel injector 2.5 mm longer than the guide cannula, connected to a 10-μL Hamilton syringe. Once the injector was inserted into the guide cannula, the drug solution (or vehicle) was infused by gently pressing the syringe’s plunger. After infusion, the injector was left in place for 10 s before removal to prevent fluid backflow. Vehicle and each NPS dose were loaded in different syringes. Yohimbine was dissolved in distilled water and administered intraperitoneally (IP) 30 min before the session.

### 2.4. Experimental procedures

#### 2.4.1. Experiment 1: Effect of NPS on open field, Elevated plus maze, and fear conditioning tasks in msP rats

Thirty-one female and twenty-two male alcohol-naïve msP rats were used in this experiment, that consisted of three consecutive tests: open field (OF), elevated plus maze (EPM) and fear conditioning (FC). Tests were adapted from previous work and conducted in a sound-attenuated room illuminated by a 30-lux red light (Borruto et al., 2021; Cippitelli et al., 2015). At least three days passed between the completion of one test and the commencement of the next. Before the beginning of the tests, rats were randomly assigned to a NPS dose (veh: 10 females + 7 males; 0.5 nmole: 10 females + 8 males; 1.0 nmole: 11 females + 7 males); the highest NPS dose was chosen based on our earlier anxiety tests conducted in male msP rats (Cannella et al., 2016). As we previously demonstrated that repeated NPS treatments do not lead to pharmacological sensitization or tolerance (Cannella et al., 2009), rats were kept in their dose groups in all tests.

##### Open Field Test.

The OF test was performed in eight OF arenas (43.18 × 43.18 cm) equipped with 16 evenly spaced infrared beams on each side to record rat location and movement. Data were acquired and analyzed by Windows-compatible Activity Monitor software (Med Associates). Rats received treatment immediately before being placed in the arena, and locomotor activity was recorded for 1h.

##### Elevated Plus Maze.

The EPM apparatus consisted of two closed and two open arms facing each other (100 cm long in total, 10 cm wide). The maze was elevated 100 cm above the floor. Rats received treatments 5 min before the test. They were individually placed in the center of the maze facing a closed arm and left exploring the maze for 5 min. Rat behavior was videorecorded and analyzed by Ethovision XT7. An entry was defined as the presence of the center of mass in an arm. The ratio of time spent in the open arms to the total test time was used as an index of anxiety.

##### Fear Conditioning.

The FC test was conducted on a 50 × 50 cm metallic grid embedded in a black wooden box, equipped with a speaker. The test lasted three days and consisted of four trials, each lasting 6 min. On day 1 (habituation trial) rats were allowed to freely explore the apparatus. On day 2 in the morning (conditioning trial) a 1-s 2.0 mA foot-shock was delivered every minute starting 59 s after the beginning of the trial (i.e. five foot-shocks in total). Each foot-shock was anticipated by a 10 s cue tone that co-terminated with the shock. Six hours later, the test trial was identical to the conditioning trial, except that the foot-shock was not delivered. Rats were treated with NPS or vehicle immediately after the end of the test trial. 24 h later, on day 3, rats entered the extinction trial, identical to the test trial. Trials were videorecorded and the amount of time spent freezing was manually scored by a trained operator blinded to treatment conditions. The ratio of time spent freezing in each trial to the total trial time was used as an index of fear conditioning.

#### 2.4.2. Experiment 2: Effect of NPS on alcohol self-administration in MsP rats

Operant alcohol self-administration (ASA) training and testing were conducted in self-administration chambers (Med Associates) equipped with a drinking reservoir (0.30 ml capacity) connected to an infusion pump, a house light, and two retractable levers, designated as the active and inactive lever, respectively. Rats were trained to self-administer 0.1 ml of 10 % alcohol (v/v) solution in daily 30-min sessions according to a fixed ratio 1 (FR1) schedule of reinforcement on the active lever. No scheduled consequence was associated to inactive lever pressing. A 5-s time-out period, during which active lever presses were not reinforced, started contingently with pump activation. The house light was illuminated during the time-out. The number of rewards, active, and inactive levers presses was recorded by Windows-compatible MedPC-5. The proper functioning of the SA program was manually verified by the operator before and after each ASA session. Consumption of the delivered alcohol solution was confirmed by the absence of residual liquid in the receptacle.

Female and male msP rats (*n* = 8/sex) were trained to ASA. After surgery, ASA was re-baselined before treatment started. The test was conducted as we previously described (Cannella et al., 2009, 2016). Rats received ICV saline injections for two consecutive days to acclimate them to the treatment procedures. On test days, rats received ICV injection of NPS (0.1, 1.0 or 2.0 nmol/rat) or its vehicle 5 min before session. This dose range included and expanded upon the one used in Experiment 1 and was selected based on earlier NPS tests on operant alcohol-seeking behavior (Cannella et al., 2016). Treatment doses were delivered in a within-subjects counterbalanced order; test sessions were repeated every fourth day until each rat had received all doses. The first day after treatment rats remained in their home cages, the second and third day they underwent ASA baseline sessions.

#### 2.4.3. Experiment 3: Effect of NPS and yohimbine combination on alcohol seeking

This experiment consisted of three phases: ASA training, extinction of alcohol seeking, and reinstatement test.

##### ASA Training:

Female and male msP rats (*n* = 9/sex) underwent ASA as described above.

##### Extinction of Alcohol Seeking:

After 20 ASA sessions, rats were subjected to 16 consecutive extinction sessions. The extinction sessions were identical to ASA sessions, except that active lever presses were reinforced by illumination of the house light and pump activation but did not result in alcohol delivery.

##### Reinstatement Test:

The day after the last extinction session, rats were treated IP with 0.625 mg/kg of yohimbine-HCl. Thirty minutes later, they received an ICV injection of NPS (0.1, 1.0, or 2.0 nmol/rat) or its vehicle. The test session, which was identical to a standard extinction session, began 5 min later. NPS doses were delivered in a within-subjects counterbalanced order. Test sessions were repeated every fourth day until each rat had received all NPS doses; rats were subjected to extinction training on intervening days.

### 2.5. Statistical analyses

Data were analyzed using appropriate between- and within-subjects ANOVAs or *t*-test. Approximation to normality of the distributions was verified using Q-Q plots of the residuals (Supplementary Figure S1) before conducting the tests. Male and female data were acquired in separate experimental sessions and were therefore analyzed separately. Statistical significance was set to conventional *p* < 0.05. Analyses were followed by Tukey’s post-hoc test when appropriate.

## 3. Results

### 3.1. Experiment 1: Effect of NPS on open field, elevated plus maze and fear conditioning tasks in msP rats

#### Open Filed Test.

**Fig. 1.**
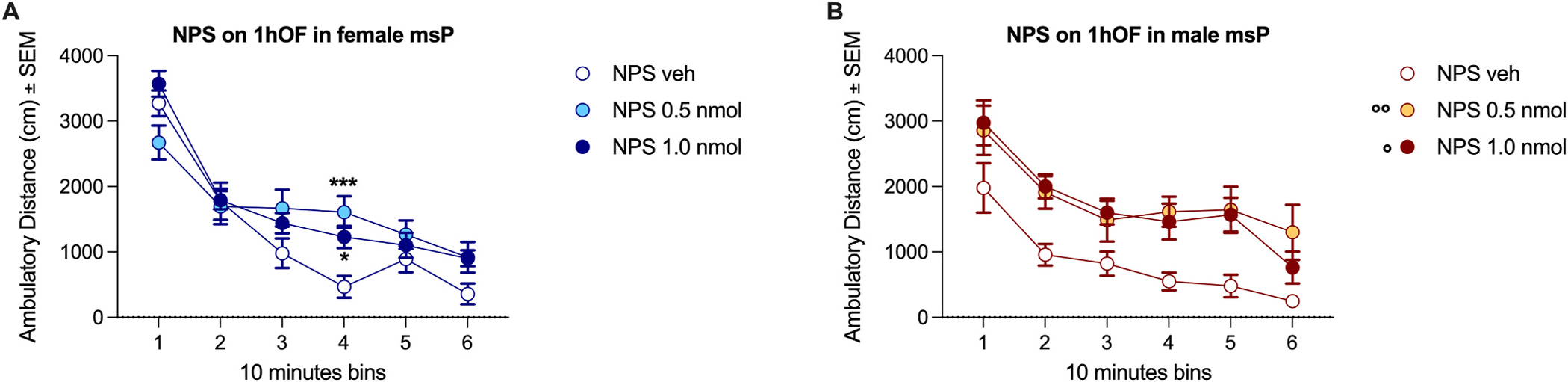
Effect of NPS on locomotor activity in msP rats. **A**) Both doses of NPS increased locomotor activity in female rats during time bin 4, i.e., between 30 and 40 min from the beginning of the test. **B**) Both doses of NPS increased locomotor activity in male rats, independently of time point. Dots and whiskers represent means ± SEM of total distance travelled during 10-min bins in the OF arena. Statistical significance: **A**) *p < 0.05 and **p < 0.01 vs. 0 in the same time bin. **B**) °p < 0.05 and °p < 0.01 vs. 0, independently of time bin.

A two-way ANOVA (NPS dose as the independent factor; 10-min time bins as the repeated measure) on distance travelled in the OF arena in female rats revealed a significant overall effect of time [F(5, 140) = 108.6, p < 0.0001, η^2^ = 0.56], no overall effect of dose [F (2, 28) = 1.66, p = 0.21, η^2^ = 0.026], but a significant dose × time interaction [F(10, 140) = 5.04, p < 0.0001, η^2^ = 0.052]. Tukey’s post hoc analysis showed that both NPS-treated groups travelled a greater distance than controls during time bin 4 (0.5 nmol, p < 0.001; 1.0 nmol, p < 0.05; Fig. 1A).

In male rats, analysis of locomotor activity revealed a significant overall effect of time [F(5, 95) = 25.8, p < 0.0001, η^2^ = 0.32] and dose [F(2, 19) = 7.18, p = 0.005, η^2^ = 0.186], but no significant dose × time interaction [F(10, 95) = 0.52, p = 0.87, η^2^ = 0.013]. This indicates that NPS increased locomotor activity independently of time. Tukey’s post hoc analysis of the main effect of dose showed that both 0.5 nmol (p < 0.01) and 1.0 nmol (p < 0.05) NPS significantly increased the distance travelled compared to vehicle (Fig. 1B).

#### Elevated Plus Maze.

**Fig. 2.**
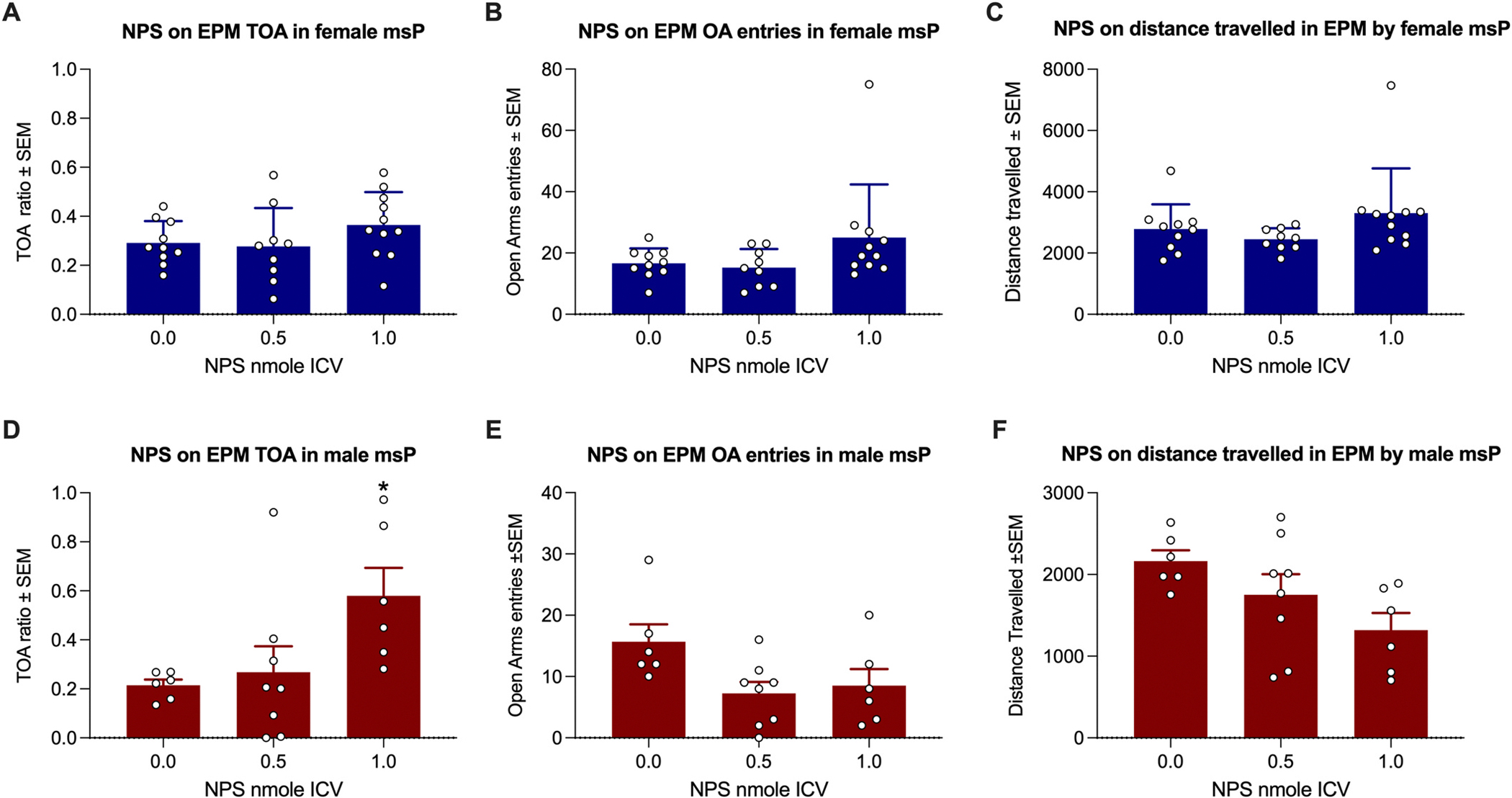
Effect of NPS on the EPM test in msP rats. **A)** NPS did not affect the time spent in the open arm of an EPM apparatus in female rats. **B-C**) Open arm entries (**B**) and total distance travelled (**C**) were not affected by NPS in female rats. **D)** 1.0 nmole of NPS significantly increased the time spent in the open arm of an EPM apparatus in male rats. **E-F)** Open arm entries (**E**) and total distance travelled (**F**) were not affected by NPS in male rats. Bars and whiskers represent means ± SEM. Statistical significance: *p < 0.05 and vs 0.0.

One-way ANOVA showed that NPS did not significantly affect the time in open arms (TOA) ratio [F(2, 27) = 1.36; *p* = 0.27, η^2^ = 0.092] (Fig. 2A), the number of open arm entries [F(2, 27) = 2.23; *p* = 0.13, η^2^ = 0.142] (Fig. 2B), or distance travelled in the EPM [F(2, 27) = 1.77; *p* = 0.19, η^2^ = 0.116] (Fig. 2C) in female msP rats.

Conversely, in males, we observed an overall effect of NPS dose on TOA ratio [F(2, 17) = 3.92; *p* = 0.039, η^2^ = 0.316], which was due to a higher TOA induced by 1 nmole of NPS (p < 0.01; Fig. 2D). The number of open arm entries [F(2, 17) = 3.43; *p* = 0.056, η^2^ = 0.287] (Fig. 2E), and the distance travelled in the EPM [F(2, 17) = 3.33; *p* = 0.06, η^2^ = 0.282] (Fig. 2F) showed non-significant trend toward a decrease. Given the relatively low *p*-values and large effect sizes in these latter two analyses, we conducted post-hoc power calculations. G*Power 3.1.9.6 indicated achieved powers of 0.66 and 0.65 for the open arm entries and distance travelled analyses, respectively.

#### Fear Conditioning.

**Fig. 3.**
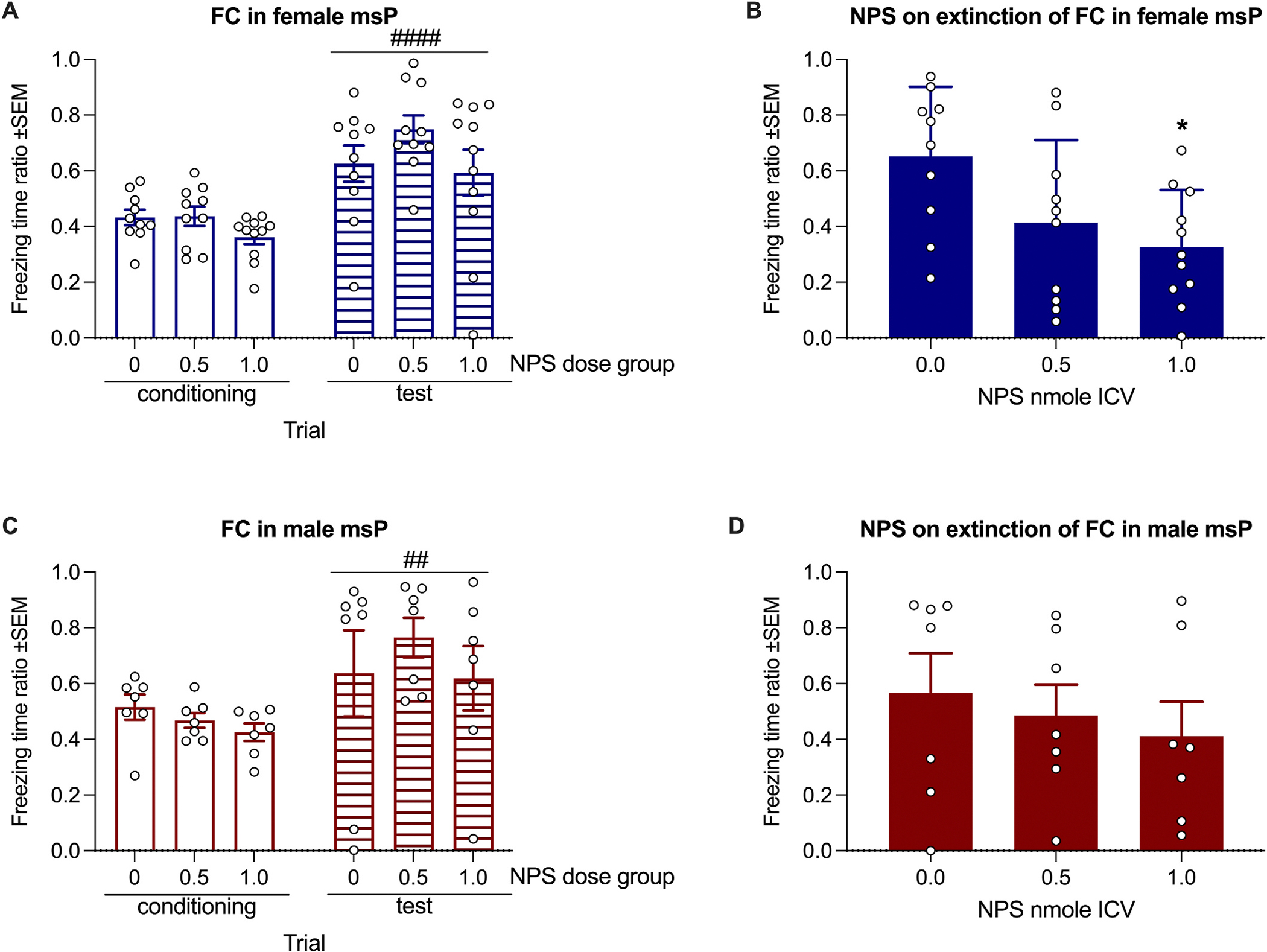
Effect of NPS administered immediately after the test trial on extinction of FC in msP rats. **A)** In female rats there was no difference in the freezing time ratio between NPS dose groups during the trials that preceded NPS treatment — i.e., conditioning and test trials — indicating a lack of group bias. In the test trial, all groups showed increased freezing compared to the conditioning trial, indicating acquisition of fear memory. **B)** In the extinction trial, the highest doses of NPS facilitated the extinction of fear memory by reducing the time spent freezing. **C)** In male rats there was no difference in the freezing time ratio between NPS dose groups during the trials that preceded NPS treatment — i.e., conditioning and test trials — indicating a lack of group bias. In the test trial, all groups showed increased freezing compared to the conditioning trial, indicating acquisition of fear memory. **D**) NPS did not affect extinction of fear conditioning in male rats. Bars and whiskers represent means ± SEM. Statistical significance: ^##^p < 0.01 and ^###^p < 0.001 vs same dose in conditioning trial, *p < 0.05 vs 0.0 dose.

A two-way ANOVA of time spent freezing by female rats during conditioning and test trials, with NPS dose group as the independent factor and trial as a repeated measure, revealed a significant main effect of trial [F(1, 28) = 46.9, *p* < 0.0001, η^2^ = 0.344], but no significant effect of group [F(2, 28) = 1.9, *p* = 0.17, η^2^ = 0.052] or group × trial interaction [F(2, 28) = 0.93, *p* = 0.41, η^2^ = 0.014]. The higher freezing observed during the test trial compared to the conditioning trial indicates successful acquisition of fear conditioning, with no group differences prior to NPS treatment (Fig. 3A). Analysis of the effect of NPS on extinction of fear conditioning found an overall effect of treatment [F(2, 28) = 4.66; *p* = 0.018, η^2^ = 0.25]. Tukey’s post hoc analysis revealed that the group treated with 1 nmol of NPS showed significantly reduced freezing compared to the vehicle group (p < 0.05; Fig. 3B).

One male (0.5 nmole) had the guide cannula blocked and was excluded from analyses. A two-way ANOVA of time spent freezing by male rats during conditioning and test trials revealed a significant main effect of trial [F(1, 18) = 10.5, *p* = 0.004, η^2^ = 0.175], but no effect of NPS dose group [F(2, 18) = 0.47, *p* = 0.63, η^2^ = 0.025] and no group × trial interaction [F(2, 18) = 0.66, *p* = 0.53, η^2^ = 0.022]. These results indicate successful acquisition of fear conditioning across all groups, with no group differences prior to NPS treatment (Fig. 3C). Analysis of freezing time during the extinction trial indicated that NPS did not affect the extinction of fear conditioning in male rats [F(2, 18) = 0.38; *p* = 0.69, η^2^ = 0.041] (Fig. 3D).

### 3.2. Experiment 2: Effect of NPS on alcohol self-administration in msP rats

**Fig. 4.**
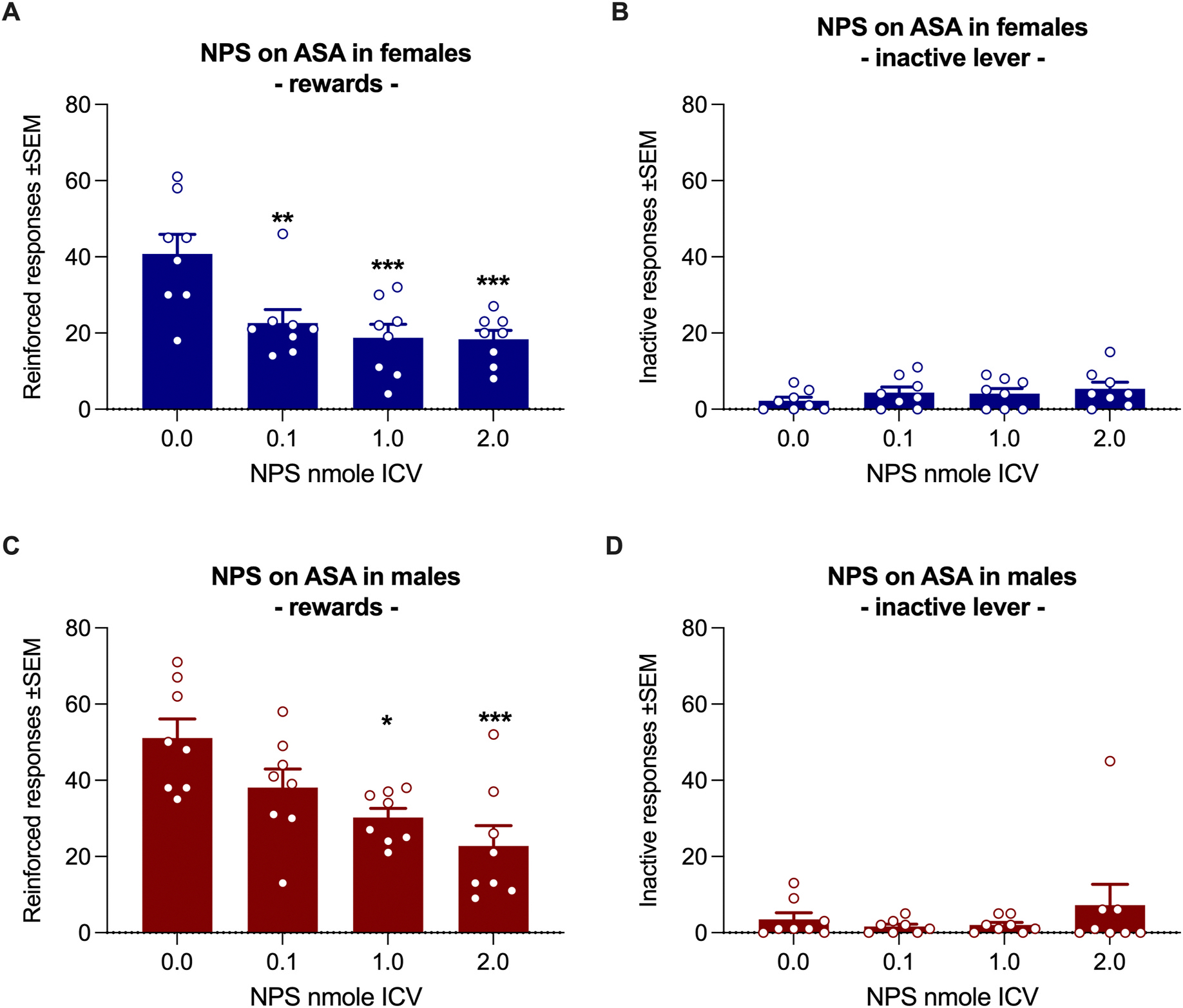
Effect of NPS on alcohol self-administration in msP rats. In female rats, all three doses of NPS reduced the number of 10 % alcohol rewards (**A**), while responses on the inactive lever were unaffected (**B**). In male rats, the intermediate and highest doses of NPS reduced the number of 10 % alcohol rewards (**C**), with no effect on responses to the inactive lever (**D**). Bars and whiskers represent the means ± SEM of reinforced active (**A, C**) and inactive (**B, D**) lever responses. Statistical significance: *p < 0.05, **p < 0.01, and ***p < 0.001 vs 0.0.

A one-way ANOVA of rewards earned by female msP rats, with NPS dose as a repeated measure, revealed a significant overall effect of treatment [F(3, 21) = 9.86, p = 0.0003, η^2^ = 0.585]. Tukey’s post hoc test showed that all three NPS doses significantly reduced alcohol rewards (0.1 nmol: p < 0.01; 1.0 and 2.0 nmol: p < 0.001; Fig. 4A). Inactive lever responses were low and unaffected by NPS treatment [F(3, 21) = 1.18, p = 0.34, η^2^ = 0.144] (Fig. 4B).

In male rats, NPS also significantly affected the number of alcohol rewards self-administered [F(3, 21) = 8.16, p = 0.0009, η^2^ = 0.538]. Tukey’s post hoc test indicated that 1.0 nmol (p < 0.05) and 2.0 nmol (p < 0.001) of NPS significantly reduced the number of rewards earned (Fig. 4C). Inactive lever responding remained unaffected by treatment [F (3, 21) = 0.98, p = 0.42, η^2^ = 0.123] (Fig. 4D).

### 3.3. Experiment 3: Effect of NPS and yohimbine combination on alcohol seeking

During the last three days of ASA, male and female rats consumed an average of 42.85 ± 2.88 and 37 ± 2.64 alcohol rewards per session, respectively.

**Fig. 5.**
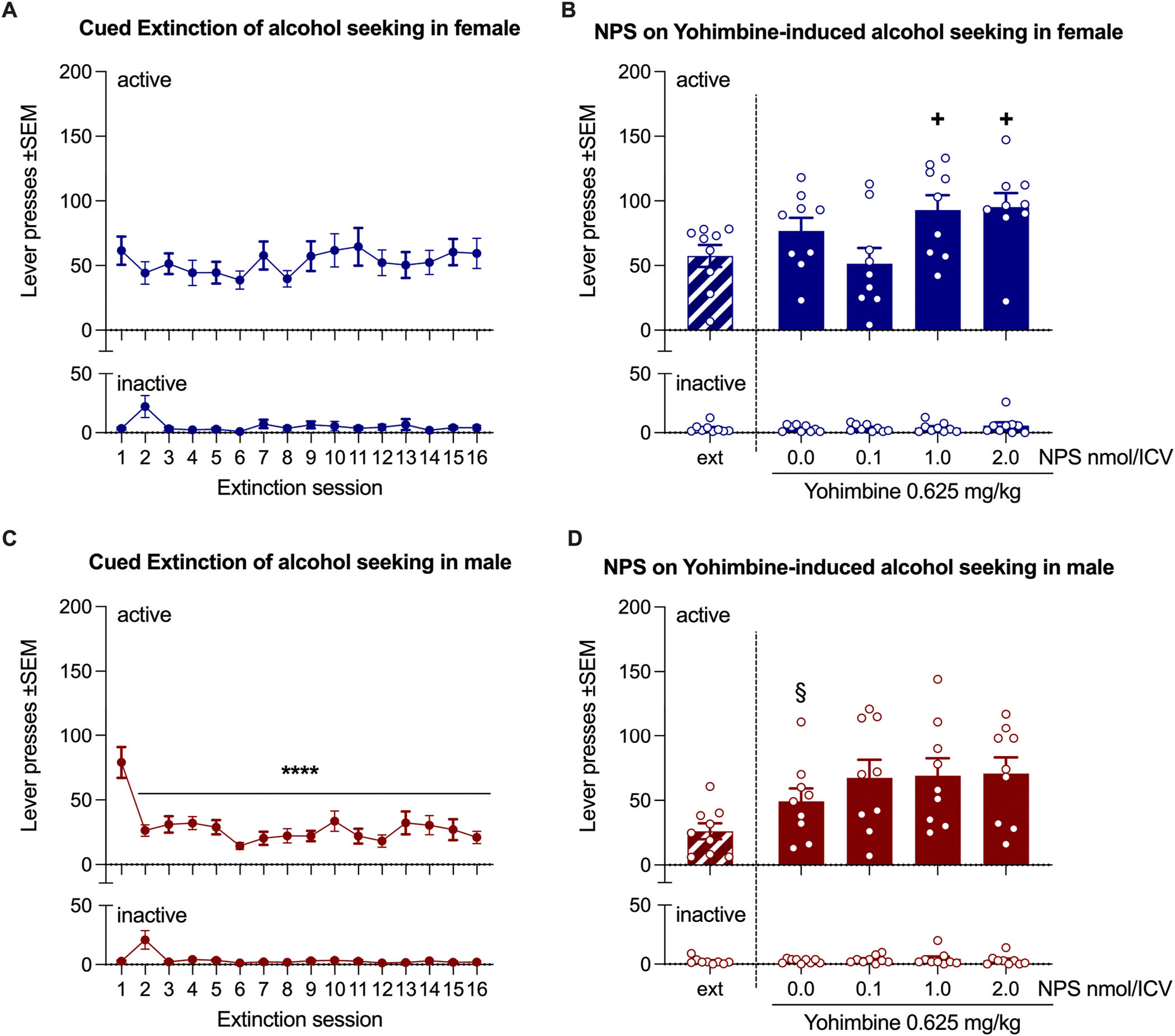
Effect of NPS and yohimbine on cued-alcohol seeking in msP rats. **A**) Female msP rats failed to extinguish alcohol-paired cued lever pressing across 16 consecutive extinction sessions. **B**) Yohimbine did not significantly increase cued lever pressing in female rats (NPS 0.0 vs extinction). When NPS was added to yohimbine, differences in active lever pressing were observed between the 0.1 nmol dose and the two higher NPS doses, but not in comparison to yohimbine + NPS vehicle. **C**) Male rats successfully extinguished alcohol-paired cued lever pressing during extinction training. **D**) Yohimbine reinstated extinguished alcohol seeking in male rats (NPS 0.0 vs extinction), and NPS did not affect yohimbine-induced alcohol seeking. The upper and lower panels display active and inactive lever responses, respectively. Bars (or dots) and whiskers represent mean ± SEM of lever responses. Statistical significance: **B**) + p < 0.05 vs NPS 0.1 nmol; **C**) ***p < 0.001 vs extinction day 1; **D**) §p < 0.001 vs extinction.

A repeated-measures one-way ANOVA of active lever responses during extinction training in female rats revealed no significant effect of time [F(15, 120) = 1.37, *p* = 0.17, η^2^ = 0.146], indicating that females failed to extinguish alcohol-seeking behavior (Fig. 5A, upper panel). Inactive lever responses were low overall, but a significant main effect of time was observed [F(15, 120) = 3.11, *p* = 0.0003, η^2^ = 0.28]. According to Tukey’s post hoc test, this effect was driven by an increase in pressing on extinction day 2 compared to all other days (Fig. 5A, lower panel). To assess the effect of yohimbine on alcohol-seeking behavior, we conducted a paired *t*-test comparing the average lever presses during the last three days of extinction to those in the yohimbine + NPS vehicle condition (extracted from the NPS treatment tests). The increase in alcohol seeking induced by yohimbine did not reach statistical significance [t(8) = 2.15, *p* = 0.064, Cohen’s *d* = 0.716]. When NPS was added to yohimbine, repeated-measures ANOVA found an overall effect of treatment [F(3, 24) = 5.15; *p* = 0.0068, η^2^ = 0.392], which, according to Tukey’s post-hoc test, was driven by differences between the NPS 0.01 nmole/ICV dose and the other two doses of NPS (p < 0.05), but not versus the vehicle (Fig. 5B upper panel). Neither yohimbine alone [t(8) = 0; p > 0.99, Cohen’s d = 0] nor the combination of NPS and yohimbine [F(3, 24) = 0.71; *p* = 0.56, η^2^ = 0.081], affected responding on the inactive lever (Fig. 5B lower panel).

Analysis of active lever responses during extinction training in male rats revealed a significant overall effect of time [F(15, 120) = 9.23, *p* < 0.0001, η^2^ = 0.536]. Tukey’s post hoc test showed that active lever pressing decreased relative to day one starting from day two (Fig. 5C, upper panel). Analysis of inactive lever responses also revealed a significant effect of time [F(15, 120) = 4.96, *p* < 0.0001, η^2^ = 0.383], which, similar to females, was due to an increase on day two (Fig. 5C, lower panel). Yohimbine reinstated alcohol-seeking behavior [t(8) = 3.46, *p* = 0.008, Cohen’s *d* = 1.154], but this effect was not altered by the addition of NPS [F(3, 24) = 1.21, *p* = 0.327, η^2^ = 0.131] (Fig. 5D, upper panel). Neither yohimbine alone [t(8) = 0.33, *p* = 0.75, Cohen’s *d* = 0.109] nor the combination of NPS and yohimbine [F(3, 24) = 0.43, *p* = 0.735, η^2^ = 0.051] affected responding on the inactive lever (Fig. 5D, lower panel).

## 4. Discussion

In this study, we aimed to characterize the role of NPS in anxiety, stress response, and alcohol seeking in msP rats, a strain showing high preference for alcohol, which they consume to mitigate their innate negative affective state (Borruto et al., 2021; Ciccocioppo et al., 2006).

Using an OF test, we demonstrated that NPS maintains its stimulatory effects (Clark et al., 2017; Xu et al., 2004) in msP rats as well. NPS was administered immediately before the OF test in rats with no prior experience in the arena. In females, the stimulatory effect was observed only during the fourth 10-min time bin, whereas in males, the stimulatory effect was present throughout the entire session. This suggests that, specifically in females, NPS was effective only after the rats had habituated to the novel arena, rather than during the early novelty-induced locomotion phase.

Previous studies have demonstrated that NPSR activation may reduce both the expression and extinction of fear memories (Bengoetxea et al., 2021; Chauveau et al., 2012; Germer et al., 2019; Jungling et al., 2008; Kawade et al., 2022). However, while these studies administered NPS right before scoring the expression or extinction of FC, here, for the first time, we administered NPS immediately after the FC test—referred to as retrieval (Bengoetxea et al., 2021; Chauveau et al., 2012; Jungling et al., 2008) or extinction training (Kawade et al., 2022) in previous studies—and assessed its effect on the extinction of FC in both males and females 24 h later. This timing corresponds to the memory consolidation phase, during which NPS enhances memory (Han et al., 2014; Okamura et al., 2011). Therefore, this approach allowed us to observe a sex-dependent effect, where NPS facilitated the extinction of fear memory in female—but not male—rats by enhancing the consolidation of fear extinction memories.

Our data corroborate the potential of the NPS system as a target for conditions associated with aberrant fear memory, such as PTSD. Together with previous studies using genetic models (Bengoetxea et al., 2021; Germer et al., 2019), our results indicate that females may also benefit from potential NPS-targeting therapies. However, the data suggest that male and female subjects may require distinct treatment approaches. For instance, a hypothetical translation of our results suggests that women might benefit more than men from a cue-assisted therapy combined with NPS agonist treatment.

Our findings revealed that NPS reduced generalized anxiety in males, as assessed in an EPM test. Notably, in an OF test, we confirmed that the anxiolytic doses of NPS retained a stimulatory effect in msPs, while the number of open arms entries and the distance travelled in the EPM showed a trend toward decreasing. This indicates that the observed increase in time spent in the open arms was not secondary to the stimulation of locomotor activity typical of NPS but rather reflected an anxiolytic effect. Importantly, post-hoc power analyses of open arm entries and distance travelled by males in the EPM indicated that these tests were underpowered to rule out Type II errors. Therefore, future studies with larger sample sizes are necessary to determine whether the anxiolytic effect of NPS in the EPM is associated with decreased locomotion.

On the one hand, EPM results confirm our previous observation of an anxiolytic effect of NPS in male msPs (Cannella et al., 2016), and on the other hand they provide a novel indication that NPS lacks this property in females. While the anxiolytic properties of NPS in males have been extensively explored and confirmed using multiple techniques across different strains and species (Clark et al., 2017; Huang et al., 2023; Rizzi et al., 2008; Tillmann et al., 2019; Vitale et al., 2008; Xu et al., 2004), the present work and that of Wegener et al. (2012) are the first to characterize this pharmacological trait of NPS in female rats. Wegener et al. (2012) examined the anxiolytic properties of NPS in three lines of rats—the Flinders Sensitive Line (FSL), Flinders Resistant Line (FRL), and Sprague–Dawley (SD) rats—and found that NPS increased EPM TOA in female FSL rats but not in FRL or SD rats. This suggests that the anxiolytic properties of NPS in females may depend on the genetic background. Notably, in Wegener’s study, female SD rats, in which NPS failed to affect the EPM TOA score, tended to be more anxious than FSL and FRL rats. Similarly, msP females are more anxious than their Wistar counterparts (Borruto et al., 2021). It is therefore tempting to speculate that in females, NPS-induced anxiolysis might be less effective in strains with higher generalized anxiety levels. However, a recent study has demonstrated that alcohol acts as an anxiolytic in an EPM setting (generalized-anxiety-like trait) in male msP but not female, whereas in females, it is effective in a fear-conditioning setting (PTSD-like trait). This suggests that generalized anxiety and PTSD-like traits may represent forms of pathological anxiety in male and female msP rats, respectively (Borruto et al., 2021). Therefore, an alternative interpretation could be that NPS exerts its anxiolytic effect only under conditions of pathological anxiety.

It should also be noted that, in a recent study conducted on female mice, Costa et al. (2024) demonstrated that the anxiolytic effect of NPS depends on the estrous phase in the marble burying test but not in the light-dark box test. The light-dark box, similar to the EPM, is considered a model of generalized anxiety, while the marble burying test is a model of obsessive-compulsive disorder (Himanshu et al., 2020). This suggests that our EPM results were not affected by the estrous cycle. However, neither did Costa et al. (2024) test EPM in their mice, nor did we probe the estrous phase of our rats; therefore, this possibility cannot be excluded. MsP rats show increased anxiety compared to their Wistar counterparts (Borruto et al., 2021; Ciccocioppo et al., 2006). Interestingly, a single nucleotide polymorphism in the **Npsr** gene — resulting in an NPSR isoform with an enhanced cAMP response to NPS stimulation — has been reported in a rat line selectively bred for high anxiety (Slattery et al., 2015). This raises the possibility that specific NPSR isoforms may have also been selected in the msP line; however, whether this actually occurred remains to be determined.

Our data indicate that the NPS system plays a role in generalized anxiety in males and in fear-stress-induced anxiety in female msP rats. MsP rats are characterized by an innate negative affective state, and the reinforcing properties of alcohol in these animals may be linked to its ability to alleviate their anxiety (Ciccocioppo et al., 2006). However, the way alcohol relieves anxiety differs between the sexes. Alcohol attenuates generalized anxiety, as measured in an EPM test, in males but not in females. Conversely, females are sensitive to the anti-stress effects of alcohol in an FC setting (Borruto et al., 2021). The sex-dependent effects of alcohol on anxiety in msP rats reported by Borruto et al. (2021) closely mirror the effects of NPS on EPM and FC observed in our study. Consistently, NPS reduced ASA in both sexes. This result confirms our previous report in males (Cannella et al., 2016) and provides a novel finding in female msP rats.

Our data suggest that NPS reduces alcohol self-administration (ASA) in msP rats through its anxiolytic properties. In previous studies, we demonstrated that NPS promotes reinstatement of cued alcohol seeking in Wistar rats by activating stress-related microcircuitries (Cannella et al., 2009; Ubaldi et al., 2016). However, this relapse-promoting effect was not observed in msP rats (Cannella et al., 2016). To extend these findings to stress-induced reinstatement, we tested the effect of NPS in combination with the widely used and translationally relevant yohimbine model of stress-induced relapse (Curley et al., 2022). In line with our previous report that in male msPs NPS acts on ASA but not on relapse models (Cannella et al., 2016), in this case NPS failed to affect yohimbine-induced reinstatement in male rats. Female msP rats did not extinguish alcohol-seeking behavior; therefore, lever responses under yohimbine treatment cannot technically be considered a reinstatement. However, in line with males, alcohol-seeking behavior was not affected by NPS.

Importantly, our yohimbine experiment was not designed to test the hypothesis that NPS may prevent yohimbine-induced alcohol seeking, as NPS was administered after, rather than before, yohimbine. Therefore, this possibility remains to be explored.

Altogether, our data demonstrate that in a model of genetic predisposition to AUD associated with negative affective states (Borruto et al., 2021; Ciccocioppo et al., 2006), activation of NPSR transmission decreases alcohol self-administration and does not affect stress-induced alcohol seeking in the absence of the primary reinforcer. This finding indicates that NPS preferentially acts on alcohol’s primary reinforcing properties. The effect of NPS in reducing alcohol intake possibly occurs by replacing the anxiolytic properties of alcohol consisting in alleviating generalized anxiety in males and PTSD like behavior in females.

In conclusion, we provide evidence that activation of NPSR has potential as a treatment for comorbid AUD and anxiety. Specifically, our data offer insights for future lines of research aimed at exploring whether greater benefits can be achieved when males present AUD comorbid with generalized anxiety, and females AUD comorbid with PTSD.

## Supplementary Material

Supplementary Material

## Data Availability

data will be made available on reasonable request to the corresponding author

## Appendix A. Supplementary data

Supplementary data to this article can be found online at https://doi.org/10.1016/j.neuropharm.2025.110598.
