# Supplementary material for: Sex-dependent role of Neuropeptide-S on anxiety, fear conditioning, and alcohol seeking in alcohol preferring rats
Source: Neuropharmacology. Author manuscript; Available in PMC 2026 May 24. (PMC13198912; doi:10.1016/j.neuropharm.2025.110598)
Supplement: Supplementary Material [file NIHMS2162064-supplement-Supplementary_Material.pdf]

SUPPLEMENTARY INFORMATION

Sex-dependent role of Neuropeptide-S on anxiety, fear conditioning, and alcohol seeking in alcohol preferring rats.

Min Li[1], Sara De Carlo[1], Laura Soverchia[1], Scott P. Runyon[2], Stewart Clark[3], Carolina L. Haass-Koffler[4], Roberto Ciccocioppo[1], Douglas J. Sheffler[5], and Nazzareno Cannella[1§].

[1]School of Pharmacy, Center for Neuroscience, Pharmacology Unit, University of Camerino, Italy.

[2] Research Triangle Institute, Center for Drug Discovery, Research Triangle Park, NC 27709, USA

[3]Department of Pharmacology and Toxicology, State University of New York at Buffalo, Buffalo, NY, USA.

[4]Department of Translational Medicine, The Scripps Research Institute, La Jolla, California, USA.

[5]Center for Alcohol and Addiction Studies, Department of Psychiatry and Human Behavior, Department of Behavioral and Social Sciences, Carney Institute for Brain Sciences, Brown University, Providence, RI, USA.

[5]Center for Therapeutics Discovery, Sanford Burnham Prebys Medical Discovery Institute, La Jolla, CA, USA.

[§] To whom correspondence should be addressed: nazzareno.cannella@unicam.it

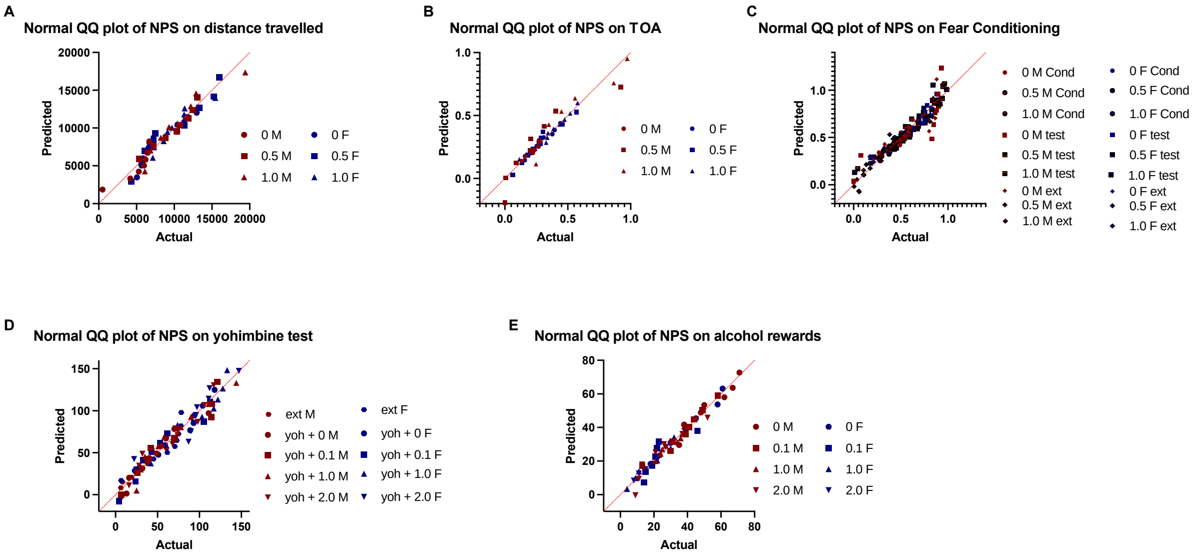

Figure S1. Q-Q plots of data used in the analyses associated to Figure 1 **(A)**, Figure 2 **(B)**, Figure 3 **(C)**, Figure 4 **(E)**, and Figure 5 **(D)**, of the main text. Legends: numbers represent NPS nmol/ICV doses; red and blue symbols represent male (M) and female (F) respectively; in panel C, Cond = Conditioning phase, test = test phase, and ext = extinction phase of fear conditioning test; In panel D, ext = extinction and yoh = yohimbine treatment.
